# Combination Antifungal Therapy for Invasive Mold Infections Among Pediatric Patients with Hematological Malignancies: Data from A Real-Life Case-Series

**DOI:** 10.20411/pai.v4i2.299

**Published:** 2019-09-05

**Authors:** Anna Maria Peri, Marta Verna, Stefano Biffi, Laura Alagna, Benedetta Longhi, Guglielmo Marco Migliorino, Sergio Foresti, Alessandra Bandera, Attilio Rovelli, Carmelo Rizzari, Andrea Gori, Antonella Colombini

**Affiliations:** 1 Infectious Diseases Unit, Department of Internal Medicine, Fondazione IRCCS Ca' Granda Ospedale Maggiore Policlinico, University of Milan, Italy; 2 Division of Infectious Diseases, Department of Internal Medicine, San Gerardo Hospital - ASST Monza, Monza, Italy; 3 Hematology-Oncology and Bone Marrow Transplantation Units, Pediatric Department, University of Milano-Bicocca, MBBM Foundation, Monza, Italy

**Keywords:** antifungal therapy, mold, hematological malignancies

## Abstract

**Background:**

Invasive mold infections in children with hematological malignancies are associated with high mortality rates. The use of combination antifungal therapy in cases with a severe clinical course is increasing, although information on the efficacy and safety of this approach is limited.

**Methods:**

We present a case series of 13 children affected by hemato-oncological disorders who received combination antifungal therapy for invasive mold infections at our center (Pediatric Hematology, San Gerardo Hospital, Monza, Italy) from 2011 to 2016, with the aim of describing their clinical characteristics, types of infections, treatment regimens, clinical outcomes, and treatment safety. Medical records were retrospectively reviewed in order to describe patients' characteristics.

**Results:**

Combination antifungal therapy included liposomal amphotericin associated with caspofungin (5/13, 38.4%), voriconazole (5/13, 38.4%), or posaconazole (3/13, 23.1%). The 12-week treatment response rate was 69.2% (6/13 patients showed complete response, 3/13 partial response). The crude mortality was 30.7% (4/13): half was related to invasive mold infections (2/13, 15.38%) and half to disease progression (2/13, 15.38%). Overall, treatment was well tolerated, and we did not observe any permanent discontinuation of antifungals due to related side effects.

**Conclusions:**

In our experience, combination antifungal therapy seems to be a safe option in immunocompromised children with invasive mold infections. Well-designed studies are needed to confirm the safety of this approach and to better understand its efficacy in the pediatric setting.

## BACKGROUND

Invasive mold infections (IMI) represent a major cause of morbidity and mortality among pediatric patients affected by hematological malignancies and are often associated with poor clinical outcomes despite the use of active antifungal drugs [1-6].

Controversies exist over the benefit of using a combination antifungal treatment (CAT) in this patient population. In particular, the attempt of improving the efficacy of the treatment is often balanced by concerns of a decreased safety due to cumulative toxicities. Evidence about the role of CAT for the treatment of IMI is scarce in the adult population and even more limited in the pediatric setting. In particular, well-designed studies on the use of CAT in immunocompromised children are lacking, and the current knowledge on this topic mainly relies on retrospective and prospective cohorts [1-6]. In this scenario, any experience of treating IMI with CAT in children may help clinicians to gain knowledge in this field and hopefully serve as a starting point for further larger studies. Hence, we report our real-life case series on the use of CAT in the pediatric setting.

## METHODS

We present a case series of IMI in children affected by hematological malignancies treated with CAT at the Hematology-Oncology and Bone Marrow Transplantation Unit, Pediatric Department, San Gerardo Hospital, Monza, Italy, from 2011 to 2016, with the aim of describing their clinical characteristics, types of infections, clinical outcomes, and treatment safety.

All patients with diagnosis of possible, probable, or proven IMI as defined by the European Organization for Research and Treatment of Cancer (EORTC) criteria were included [7]. Medical records were retrospectively reviewed to describe patients' characteristics, outcomes, and treatment safety.

For each patient we recorded the clinical outcome 12 weeks after diagnosis of IMI: among living patients we distinguished those with complete response to treatment (full recovery) and those with partial response (major improvement of symptoms and radiologic findings).

## RESULTS

During the study period (2011-2016) 13 cases of IMI in pediatric patients affected by hematological malignancies were treated with CAT at our Center. Eight of 13 patients (61.5%) were males and the mean age was 10 years (3 months-16 years) For demographic characteristics of patients see Table 1.

**Table 1.** Demographics and characteristics of hematological malignancies of 13 patients with invasive mold infections

Patient	Gender and Age (years)	Ethnicity	Haematological Disease	Time of Occurrence	Haematological Treatment	Neutropenia (N/mm^3^)	GVHD	Steroid (prednisone equivalent)
1	M, 9	White	Relapsed ALL	Induction Therapy	CT	< 100	No	> 2 mg/kg day
2	M, 14	White	Relapsed ALL	allo-SCT (day+10)	CyA, MTX, ATG	< 100	No	No
3	F, 5	White	ALL front line	Induction Therapy	CT	< 500	No	> 2 mg/kg day
4	F, 8	White	ALL front line	Induction Therapy	CT	< 100	No	> 2 mg/kg day
5	M, 13	White	ALL front line	Induction Therapy	CT	< 100	No	> 2 mg/kg day
6	M, 16	White	ALL front line	Induction Ttherapy	CT	> 500	No	> 2 mg/kg day
7	M, 12	White	NHL	relapse/salvage	CT	<100	No	> 2 mg/kg day
8	F, 3	White	Relapsed ALL	relapse/salvage	CT	< 100	No	< 2 mg/kg day
9	M, 15	White	Relapsed AML	relapse/salvage	CT	< 100	No	> 2 mg/kg day
10	M, 16	Asian	CML	allo-SCT (day+200)	MMF, PDN	500-1000	Yes	> 2 mg/kg day
11	F, 12	White	ALL front line	Induction Therapy	CT	< 100	No	> 2 mg/kg day
12	M, 10	Asian	Relapsed NHL	allo-SCT (day+120)	CyA, PDN	< 500	No	< 2 mg/kg day
13	F, 90 days	African	HLH	Induction Therapy	DXM, VP16, ATG	< 500	No	> 2 mg/kg day

Abbreviations: M = Male; F = Female; ALL = Acute Lymphoid Leukemia; NHL = Non-Hodgkin Lymphoma; AML = Acute Myeloid Leukemia; CML = chronic myeloid leukemia; HLH = haemophagocitic hystiocitosis; SCT = Stem Cells Transplantation; CyA = Cyclosporine A; MTX = methotrexate; ATG = anti-thymocyte globulin; CT = Chemotherapy; MMF = mycofenolate mofetil; PDN = prednisone; DXM = dexamethasone; VP16 = etoposide; N = neutrophils; GVHD = Graft Versus Host Disease

### Invasive Mold Infections and Disease Site Involvement

Five patients (38.5%) met EORTC criteria for proven IMI. In particular, *Absidia Corymbifera* and another untyped Mucorales grew on the orbital biopsies of 2 patients with rhino-orbital-cerebral mucormycosis while *Aspergillus flavus* grew on the orbital biopsy of 1 patient with rhino-cerebral and pulmonary aspergillosis; *Fusarium subglutinans* was isolated from blood cultures of a fourth patient with disseminated fusariosis with cutaneous involvement. A fifth patient had hyphae on a liver biopsy establishing the diagnosis of proven IMI, probable Aspergillosis (no results from cultures were available) with hepatosplenic and pulmonary involvement.

Five more patients had probable pulmonary aspergillosis, documented by Galactomannan antigen assay and compatible clinical-radiological signs. These patients all had pulmonary disease with 3 of them also having central nervous system (CNS) involvement and 1 having liver involvement.

The remaining 3 patients had possible pulmonary aspergillosis with 1 also having hepatosplenic involvement.

Of note, the diagnosis of probable or possible hepatosplenic involvement was established by imaging. Characteristics of IMI are summarised in Table 2.

**Table 2.** Characteristics of invasive mold infections (IMI) and patients' outcome.

Patient	EORTC Classification	Species	Site of IMI	Concurrent Infections	Combination Therapy	Outcome and 12-weeks Response
1	Possible aspergillosis	-	lung, liver, spleen	coronavirus 229E/NL63	L-AMB + caspofungin	Favourable (CR)
2	Probable aspergillosis	-	lung	BSI S.mitis/R. mucilaginosa	L-AMB + caspofungin	Favourable (PR)
3	Probable aspergillosis	-	lung, CNS	RSV B	L-AMB + voriconazole	Failure, death due to IMI
4	Proven IMI (probable aspergillosis)	-	lung, liver, spleen	No	L-AMB + caspofungin	Favourable (CR)
5	Probable aspergillosis	-	lung, CNS, liver	BSI S. Epidermidis	L-AMB + voriconazole	Favourable (PR)
6	Proven mucormycosis	Absidia corymbifera	rinocerebral	No	L-AMB + posaconazole	Favourable (CR)
7	Proven mucormycosis	Untyped mucorales	rhino-orbito-cerebral	No	L-AMB + posaconazole	Favourable (CR)
8	Proven fusariosis	Fusarium subglutinans	BSI, skin	rinovirus A/B/C	L-AMB + voriconazole	Favourable (CR)
9	Possible aspergillosis	-	lung	No	L-AMB + posaconazole	Failure, death due to HD
10	Probable aspergillosis	-	lung, CNS	CMV viremia	L-AMB + voriconazole	Failure, death due to IMI
11	Probable aspergillosis	-	lung	BSI P. aeruginosa	L-AMB + caspofungin	Favourable (PR)
12	Possible aspergillosis	-	lung	VZV Zoster C5-C7	L-AMB + voriconazole	Favourable (CR)
13	Proven aspergillosis	Aspergillus flavus	lung, rhinocerebral	K.pneumoniae UTI	L-AMB + caspofungin	Failure, death due to HD

Abbreviations: EORTC: European Organization for Research and Treatment of Cancer IMI = Invasive Mold Infections; CNS = Central Nervous System; BSI = Bloodstream Infection; RSV = Respiratory Syncitial Virus; CMV = Cytomegalovirus; VZV = Varicella Zoster Virus; UTI = Urinary Tract Infection; L-AMB = liposomal amphotericin B; CR = Complete Response; PR = Partial Response; HD = Hematological Disease.

### Hematological Malignancies

Ten patients developed IMI while receiving chemotherapy and 3 following hematopoietic stem cell transplantation (HSCT).

Among patients receiving chemotherapy, 7/10 were affected by acute lymphoblastic leukemia (ALL) (of whom 5 in front-line therapy and 2 in first-relapse), 1/10 was affected by first-relapsed acute myeloid leukemia (AML), 1/10 by first-relapsed non-Hodgkin lymphoma (NHL) and 1/10 by haemophagocitic lymphohistiocytosis (HLH) in front-line treatment.

All were in the most intensive phases of chemotherapy (6 in the induction and 4 in the re-induction phase), essentially with anthracyclines, vincristine, steroids, antimetabolites, and alkylating agents.

In the second group patients received HSCT for relapsed NHL, relapsed ALL, and chronic myeloid leukemia. All of them received a myeloablative conditioning regimen. One patient had liver graft-versus-host-disease and was receiving treatment with cyclosporine, mycophenolate, and steroids.

All patients were neutropenic at the time of the diagnosis of IMI: in particular 8/13 patients (62%) had neutrophil count < 100/μL, 3/13 patients (23%) had neutrophil count within 100-500/μL and 2/13 patients within 501-1000/μL. Severe neutropenia (defined as neutrophil count < 500/mm^3^) preceding the diagnosis of IMI lasted on average 10.9 days (5-26 days).

Moreover, 12/13 patients were receiving steroids (7 prednisolone, 2 dexamethasone, 3 methylprednisolone), of whom 83% were receiving high doses (prednisone equivalent > 2mg/Kg/daily). All transplanted patients were also undergoing prophylaxis with cyclosporine. According to internal guidelines, 7/13 patients were receiving antifungal prophylaxis for high-risk underlying disease, 4 with liposomal amphotericin (L-AMB), 2 with fluconazole, one with posaconazole. Characteristics of hematological malignancies are summarised in Table 1

### Treatment

Ten out of 13 patients received CAT as salvage treatment while the remaining 3/13 received CAT as first-line approach: in the latter group, 2 were undergoing prophylaxis with L-AMB twice/weekly and one with fluconazole (6-10 mg/Kg/daily) when diagnosis of IMI was established.

Combination regimens included L-AMB associated with caspofungin (5/13, 38.4%), posaconazole (3/13, 23.1%), and voriconazole (5/13, 38.4%). Ten patients received L-AMB at 3-5 mg/Kg/daily while 3 patients with CNS involvement received 5-10 mg/Kg/daily. Caspofungin was used at the dosage of 50 mg/m2/daily while the dosage of posaconazole and voriconazole was titrated according to their plasmatic concentrations (Therapeutic Drug Monitoring, TDM). Caspofungin was chosen as the second antifungal agent in 5 patients with invasive aspergillosis (IA), while posaconazole was given to 2 patients with rhino-orbital-cerebral mucormycosis, and to 1 patient with possible IA who was already receiving L-AMB prophylaxis.; voriconazole was chosen as the second agent in 3 patients with CNS aspergillosis, in 1 patient with pulmonary aspergillosis and in 1 patient with invasive fusariosis. The mean duration of CAT was 40 days (5-98 days). Maxillo-facial surgery was required in cases of rhino-orbital-cerebral mucormycosis and aspergillosis. For treatment see Table 3.

**Table 3.** Combination antifungal therapy regimens.

Patient	Monotherapy Before Combination	Duration of Monotherapy (Days)	Combination Therapy	Duration of Combination Therapy (Days)	Maintainance Therapy	Duration of Maintainance Therapy (Months)	Surgery
1	L-AMB	11	L-AMB + caspofungin	37	Voriconazole; L-AMB posaconazole^§^	12	No
2	L-AMB	12	L-AMB + caspofungin	48	L-AMB	> 18	No
3	L-AMB	2	L-AMB + voriconazole	11	[NA]	[NA]	No
4	L-AMB	21	L-AMB + caspofungin	41	Voriconazole	10	No
5	L-AMB	11	L-AMB + voriconazole	49	Voriconazole	15	No
6	voriconazole	3	L-AMB + posaconazole	92	Posaconazole	48	Yes
7	L-AMB	12	L-AMB + posaconazole	98	L-AMB posaconazole^§^	4	Yes
8	L-AMB	6	L-AMB + voriconazole	10	Voriconazole	1, 5	No
9	No	-	L-AMB + posaconazole	28	[NA]	[NA]	No
10	No	-	L-AMB + voriconazole	5	[NA]	[NA]	No
11	No	-	L-AMB + caspofungin	36	Voriconazole	12	No
12	L-AMB	24	L-AMB + voriconazole	17	Voriconazole	48	No
13	L-AMB	unknown	L-AMB + caspofungin	50	[NA]	[NA]	Yes

(*) Including laboratory investigations

§ consecutive regimens

Abbreviations: L-AMB = liposomal amphotericin B; NA = Non Applicable.

### Outcome

We recorded the outcome 12 weeks after the diagnosis of IMI. Overall mortality was 30.7% (4/13). Two deaths were related to progression of CNS aspergillosis and 2 to progression of the underlying malignancy. The 12-week overall response was 69.2%: 6 patients showed complete response (one reporting monocular blindness after surgical curettage of the orbit), while 3 showed partial response (see Table 2).

### Safety

Toxicities during CAT were classified according to Common Terminology Criteria for Adverse Events [8]. Overall, CAT was well tolerated. Seven patients reported transitory altered liver tests, including bilirubin, transaminases, and gamma-glutamyltransferase (GGT). In particular 2 patients reported a grade 1 increase of liver markers (both of GGT and one of transaminases), 2 patients reported a grade 2 increase of bilirubin with 1 of them also reporting a grade 2 increase of GGT and the other reporting a grade 2 increase of transaminases. The remaining 3 patients reported a grade 3 increase of GGT with 2 of them also reporting a grade 3 increase of transaminases.

Moreover, 6 patients reported acute kidney injury (AKI) and 6 hypokalemia. Markers of liver dysfunction were high in 4 patients treated with the combination L-AMB + voriconazole and in 3 patients treated with the combination L-AMB + caspofungin, of note, among these patients 2 also had hepatic involvement by the fungal infection.

In 1 case AKI led to L-AMB dose reduction, in another patient altered liver tests led to temporary discontinuation of CAT that was safely reintroduced 24 hours later, after improvement of the hepatic function. No definitive discontinuation occurred (see Table 4).

**Table 4.** Toxicities attributed to combination antifungal therapy.

Patient	Hepatobiliary Toxicity*	Renal Toxicity*	Treatment Discontinuation or Modification
1	Liver test increase grade 2		
2	Liver test increase grade 3	AKI grade 1	
3	Liver test increase grade 3		CAT temporary discontinuation (24 hours)
4	Liver test increase grade 3	Hypokalemia grade 2	
5		AKI grade 2 Hypokalemia grade 2	
6		AKI grade 2	
7	Liver test increase grade 2	AKI grade 2	L-AMB dose reduction
8		Hypokalemia grade 2	
9	Liver test increase grade 1	Hypokalemia grade 2	
10		AKI grade 1 Hypokalemia grade 2	
11		Hypokalemia grade 2	
12	Liver test increase grade 1	AKI grade 1	
13			

Abbreviations: L-AMB = liposomal amphotericin B; AKI = Acute Kidney Injury; CAT = combination antifungal therapy.

## DISCUSSION AND CONCLUSIONS

Despite an improvement in the outcome of IMI in children with hematological malignancies having been reported by recent studies compared to studies from the last few decades [9-11], mortality is still high in this patient population. In particular, recent studies on children with hemato-oncological disorders and IMI treated with different regimens report crude mortality rates ranging from 30% to 52.5% [1-6], in line with our case series, in which the overall death rate was 30.7%.

CAT is often chosen as a strategy to improve the outcome in those cases of IMI with severe clinical course: its main goal is to widen the spectrum of therapy, to lower the risk of resistance and to exploit the synergism between some drugs.

Few randomized controlled studies have been published on adult patients assessing the role of CAT compared to monotherapy in the clinical setting, and there is still very little evidence about the efficacy and safety of this approach [12]. If evidence is limited in the adult population, it is even scarcer in the pediatric setting.

The Infectious Diseases Society of America (IDSA) and European Society of Clinical Microbiology and Infectious Diseases (ESCMID) guidelines still recommend monotherapy for the majority of IMI. Combination therapy with echinocandins + L-AMB or azoles are listed as an alternative in salvage therapy for IA while the association of L-AMB with posaconazole or caspofungin is regarded as an option for refractory mucormycosis [13-15].

Despite the lack of well-designed trials assessing the efficacy and safety of this approach, combination antifungal regimens are widely used in real-life pediatric settings as reported by several studies. Monocentric surveys on IMI in immunocompromised children reported the use of combination therapy in a percentage of cases ranging from 32% to 92% [2-4]. A multicenter cohort study performed by Burgos et al, involving 6 US pediatric centers, reported the use of combination regimens in up to 80% of IMI [1]. Moreover, a multicenter international prospective cohort study on mucormycosis by Pana *et al* reported the use of first-line combination therapy in 53% of cases [6].

Similar to our findings, several pediatric series report the use of CAT mainly as salvage treatment [5, 16-19], but also as first-line, especially in patients receiving anti-mold prophylaxis [5].

In a recent Italian multicenter study on 127 invasive fungal infections in hemato-oncological pediatric patients, up to 45/126 (36%) episodes were treated with antifungal combinations as initial therapy and the author speculates that this choice was driven by the more severe clinical conditions of these patients [19]. Furthermore, in the international multicenter observational cohort study by Wattier et al on 131 IMI in children affected by hematological neoplasms, first-line combination therapy was used in 53% of cases and, similar to our findings, it was mainly used in patients who were already receiving anti-mold prophylaxis possibly due to concerns about prophylaxis-induced resistance [5].

Overall response to combination therapy in our case series was 69.2% with an IMI associated mortality of 15.4%, and no definitive discontinuations related to toxicity occurred.

Data from the pediatric literature on CAT report similar rates of favorable responses and toxicity, although the heterogeneity of patient populations and antifungal regimens of such studies, together with different underlying neoplasms and fungal infections included, prevent from drawing any evidence about the superiority of CAT over monotherapy in relation to outcome and safety [3, 16-18]. Indeed, outcomes reported in our case series are not different from outcomes reported in the literature in children treated with monotherapy, and overall there is paucity of well-designed studies aimed at comparing mortality in pediatric patients treated with monotherapy versus combination therapy.

In particular, only 1 prospective study from Wattier et al compared outcomes and safety of 131 children with IMI (75% IA) treated with CAT (70/131, 53%) or monotherapy (61/131, 47%) [5]. In that study, no favorable association between CAT and outcome was found; conversely, CAT was associated with increased toxicity. However, the study had several limitations, indeed acknowledged by the authors, in particular the heterogeneity of CAT. Furthermore, the ineffectiveness of CAT on the outcome was hampered by several confounding factors (with the more severely ill patients being more likely to receive more aggressive therapy but also being more likely to experience poor outcomes), and the association of CAT with toxicity could be a result of the underlying severity of the illnesses.

In our center, the combination of L-AMB with caspofungin was used for IA as the main CAT. This combination is synergic *in vitro* [20] and showed promising clinical results among both adults [12] and children [18]. All but 1 patient (4/5) treated with this association survived. The fifth died due to the progression of the underlying malignancy; of note, this patient was the only one in this group to have aspergillosis with rhino-cerebral involvement.

Conflicting results are available in the literature about the association of L-AMB with azoles. Some *in vitro* studies have reported antagonism between the 2 drugs [21] while this combination has proven to be effective and associated with improved outcomes in animal models of CNS aspergillosis [22-24]. Although evidence is controversial, many real-life studies report the use of this combination in clinical practice in the pediatric setting [4, 5, 19]. In our case series, the combination of L-AMB with voriconazole was mainly used to treat CNS aspergillosis. Among these patients, mortality rate was high: Of note, CNS aspergillosis is historically associated with poor outcome [13] and studies are needed to establish the best treatment.

The intrinsic resistance of *Fusarium* species to most antifungals leads to high mortality rates in patients with invasive fusariosis. Despite the concerns about *in vitro* antagonism between polyenes and azoles, some studies have shown *in vitro* synergism of voriconazole and L-AMB against *Fusarium* [25]. Based on such studies and on few clinical reports of success in the treatment of invasive fusariosis with the association of L-AMB with voriconazole, some authors support CAT for fusariosis [26-29]. In this case series 1 patient with invasive fusariosis was successfully treated with L-AMB associated with voriconazole.

Lastly, the association of L-AMB with caspofungin or posaconazole is associated with encouraging results in the salvage treatment of mucormycosis [14, 15]. We have described 2 cases of mucormycosis with rhino-cerebral and orbital involvement successfully treated with L-AMB in association with posaconazole and surgery. A recent study from Greece by Pana et al reported the use of CAT in 26/63 children with invasive mucormycosis [6]; however, the impact of CAT on survival could not be estimated due to the small patient population.

In the present age, cases of IMI in immunocompromised children affected by hematological malignancies are still associated with high morbidity and mortality. Through our case series we report our real-life experience in treating IMI with CAT at our pediatric center. Given the merely descriptive purpose of our report which lacks a statistical analysis, we are aware that our work will not provide enough evidence for further recommendations about efficacy and safety of CAT in children with hemato-oncological malignancies affected by IMI. However, given the scarcity of data about this topic in the literature together with the rarity of such infections in this patient population yet associated with a severe prognosis, we believe that even isolated experiences from single-center institutions can add useful knowledge to this field.

While waiting for well-designed controlled studies, CAT might be regarded as a possible approach for IMI in children with hemato-oncological diseases; in particular, the use of CAT could be considered early when prognosis is not favorable.

## ETHICS APPROVAL AND CONSENT

This study has been performed in accordance with the Declaration of Helsinki. Parental/guardian informed consent, previously approved by our Ethical Committee, was obtained for all patients for the use of anonymous data for research purposes and publication.
